# Variation in Cold-Related Mortality in England Since the Introduction of the Cold Weather Plan: Which Areas Have the Greatest Unmet Needs?

**DOI:** 10.3390/ijerph15112588

**Published:** 2018-11-19

**Authors:** Peninah Murage, Shakoor Hajat, Angie Bone

**Affiliations:** 1 Department of Public Health, Environment and Society, London School of Hygiene and Tropical Medicine, 15–17 Tavistock Place, London, WC1H 9SH, UK; shakoor.hajat@lshtm.ac.uk; 2 European Centre for Environment and Human Health, University of Exeter College of Medicine and Health, Heavitree Road, Exeter, EX1 2LU, UK; angie.bone19@gmail.com

**Keywords:** cold weather, public health intervention, mortality, spatial variation

## Abstract

The Cold Weather Plan (CWP) in England was introduced to prevent the adverse health effects of cold weather; however, its impact is currently unknown. This study characterizes cold-related mortality and fuel poverty at STP (Sustainability and Transformation Partnership) level, and assesses changes in cold risk since the introduction of the CWP. Time series regression was used to estimate mortality risk for up to 28 days following exposure. Area level fuel poverty was used to indicate mitigation against cold exposure and mapped alongside area level risk. We found STP variations in mortality risk, ranging from 1.74, 1.44–2.09 (relative risk (RR), 95% CI) in Somerset, to 1.19, 1.01–1.40 in Cambridge and Peterborough. Following the introduction of the CWP, national-level mortality risk declined significantly in those aged 0–64 (1.34, 1.23–1.45, to 1.09, 1.00–1.19), but increased significantly among those aged 75+ (1.36, 1.28–1.44, to 1.58, 1.47–1.70) and for respiratory conditions (1.78, 1.56–2.02, to 2.4, 2.10–2.79). We show how spatial variation in cold mortality risk has increased since the introduction of the CWP, which may reflect differences in implementation of the plan. Combining risk with fuel poverty information identifies 14 STPs with the greatest need to address the cold effect, and that would gain most from enhanced CWP activity or additional intervention measures.

## 1. Introduction

Although significant gains were made in reducing cold-related health burdens throughout much of the last century [1], there is evidence that risk reductions have plateaued in more recent periods [2]. As such, cold-related health remains an important public health issue, especially in the UK. where burdens remain higher than many of its European neighbors [3]. Whilst mortality is the most extreme outcome of cold exposure, other associated outcomes, such as increase in GP (general practitioner) consultations and hospital admissions, also result in considerable burden on the health services. The provision of health services in England is overseen by the National Health Service (NHS), which provides universal and free health coverage for all UK residents. The cost that is associated with inadequately-heated private housing is estimated to be £848 million [4]. The full cost to society, including spending by social services, loss of productivity, and workplace absenteeism is not known. 

In recognition of these impacts and recent severe winters, the Cold Weather Plan (CWP) for England was introduced in 2011 in order to prevent the harmful effects of cold weather, and to reduce the burden on health and social care systems [5]. The CWP provides a national framework for guiding and coordinating the approach of public agencies, communities, and individuals who work to raise awareness and initiate action at the local level. CWP thereby encompasses all efforts on mitigating against cold weather effects, such as the National Energy Action referral scheme and government Winter Fuel and Cold Weather Payments, as well as local and regional schemes such as Making Every Contact Count (MECC) Programme in the Yorkshire and the Humber region, and many others [5]. Suggested activities are categorized into four levels of increasing severity: Level 0 involves year-round planning to build resilience and includes activities on housing and energy efficiency measures and influencing behavior change [5]. Level 1 is centered on winter preparedness and involves activities such as issuing influenza vaccinations. The higher levels 2–4 are reactive in nature and are mostly relevant to emergency planners [5]. The activities in these levels range from sign-posting the most vulnerable individuals to issuing major alerts in the instance of severe cold-weather [5]. A study evaluating the CWP implementation identified conditions during which local level implementation may be cost-effective, and provided some assurance that targeting vulnerable populations in the CWP implementation may reduce cold-related burdens [6]. However, that study was based on mathematical modelling due to lack of post-intervention data. There is presently no epidemiological evidence on how the CWP impacts health outcomes in England [7].

Previous work has highlighted important regional differences in cold risk in England that are likely to be partly explained by differences in socio-economic factors, including fuel poverty rates [8]. Variations in risk may have been exacerbated in recent years by differences in the level of implementation of the CWP. Furthermore, as there are likely to be important variations in cold risk at a sub-regional level, the effectiveness of the CWP could be enhanced if temperature–health relationships were better characterized at a small-area level to allow for more targeted implementation. 

This study characterizes the relationship between low ambient temperature and mortality in England for the most recent time-period, and assesses whether there have been any clear changes in risk in the years since the introduction of the CWP. The analysis is undertaken at both the national level and at the level of the 44 newly formed geographical units STPs (Sustainability and Transformation Partnerships). STPs are local partnerships between health services and local councils with an average population of 1.2 million, and are now considered central to delivering the transformation objectives detailed in the “NHS Five Year Forward View” [9]. A King’s Fund study reported variations in developing STP priorities, such as in addressing housing and environmental issues [10], which may be related to the extent of historical collaborations between the NHS and local government [10]. Whilst the effect of such variations is not yet apparent, local decision-making may have consequences on preventing the health effects of cold exposure. For instance, the higher winter mortality in England has been attributed to poorly insulated housing [11], meaning some mortality may be avoided by improving the thermal efficiency of housing [3,11].

We compared the mortality risk associated with cold exposure in the periods before and after CWP implementation. We also examined area variations in mortality risk and levels of fuel poverty, and used these two indicators to highlight areas where risk is worse than the national median, and where the proportion of fuel poor households is higher than average. It is likely that such areas may not meet the need to prevent the harmful effects of cold weather on health and are thereby likely to have the most significant gains from intervention measures.

## 2. Methods

STP daily mean temperatures were computed from the UK Meteorological Office hourly temperature data. This was done by identifying weather stations within each STP, and estimating a composite temperature series from stations that recorded data on at least 75% of days during the study period (2007–2015); missing data were imputed using a previously published methodology [12,13]. National level data on mean daily temperature across the study period were obtained from the Central England Temperature (CET) dataset [14].

Office for National Statistics mortality data were also obtained for the same study period, and each record was assigned to the corresponding STP using postcode information. Cause specific mortality was defined for cardiovascular (ICD10 I00-I99) and respiratory (ICD J00-J99) conditions. National level mortality was grouped using the following age groups: 0–64, 65–74, 75+ years. The data were aggregated by date of death to create a time series of a daily death count for the study period.

Fuel poverty statistics were used to indicate the extent of local area mitigation against the adverse effects of cold exposure. The fuel poverty indicator simultaneously measures the proportion of households whose fuel costs are above the national median, and whose residual income would fall below the poverty line after paying for their fuel consumption [15]. The proportion of fuel poor households per STP was computed by aggregating the 2015 LSOA (Lower Super Output Area) statistics.

### 2.1. Statistical and Spatial Analysis 

The analysis was restricted to winter months (November to March) when cold exposure is expected to have the highest effect on health. The analysis involved the following two stages:

#### Stage 1: Quantifying the Cold Effect on Mortality by Disease and Age Group

Time series, quasi-Poisson regression models were used to estimate temperature–mortality associations during the study period, 2007–2015, and during the periods preceding and following CWP introduction, 2007–2010 and 2012–2015, respectively. These associations were reported as relative risks (RRs) between two threshold temperatures (first temperature percentile and maximum temperature). In the sensitivity analysis, we also estimated the relative risk of death using temperature values that are common to all STPs, by comparing the risk between 0 °C and 13 °C for every STP. These temperature values were selected based on the STPs’ temperature distribution and indicate points of minimum (13 °C) and maximum mortality (0 °C). Further, the relative risk by disease and age-groups was estimated at an England level; this sub-group analysis was not possible at the level of STP due to small numbers.

The analysis was conducted on R version 3.3.2 (R Foundation for Statistical Computing, Vienna, Austria), using the dlnm (distributed lag non-linear model) package that simultaneously models the non-linear and delayed effects between temperature and mortality [16]. The delayed effect represents temporal change in mortality after cold exposure, and estimates the distribution of immediate and delayed effects of cold on health that accumulate across a specified time period (lags) [17]; a lag of 28 days was specified in this study [18]. The cold effect was estimated using a cross basis, and spline functions were used to flexibly model the relationship between temperature and mortality, and the lag-distribution. Detailed information of how the cross-basis operates within the dlnm package has been previously published [16,17,19]. 

The models were adjusted for seasonal variation and long-time term trend by fitting a natural cubic spline on the day of year and time variables, respectively, an indicator for the day of the week. The argument “group” in the dlnm package was used to define groups of observations representing multiple and independent series based on winter months [19].

To estimate the national and STP level relative risk (within a 95% CI), we used the threshold function within dlm (distributed lag linear model), and assumed that the cold effect on mortality was null above the cold threshold (highest temperature), and increased linearly below this threshold. We thereafter calculated the mortality burden attributable to cold temperature by applying a technique suggested for dlnm [20]; which gives the avoidable deaths at the first temperature percentile using a counterfactual condition (highest temperature). 

#### Stage 2: Identifying Areas with the Greatest Need for Addressing Cold Risk

ArcGIS software (ESRI, Redlands, CA, USA) was used to map the STP level mortality risk, the change in risk in comparison with the national median, and the proportion of households in fuel poverty in 2015. STPs with the greatest need to address cold-related mortality were identified as those with an unfavorable change in mortality risk (compared to the median STP), and with higher than the national average proportion of fuel poverty. Additionally, variation in risk between STPs was measured using the I-squared statistic [21]. 

## 3. Results

The average number of weather stations per STP was 20 and this ranged from 6 to 66 stations depending on the size of the STP. A figure showing the location and relative sizes of these STPs has been added in the supplementary material (Figure S1).

**Figure 1:**
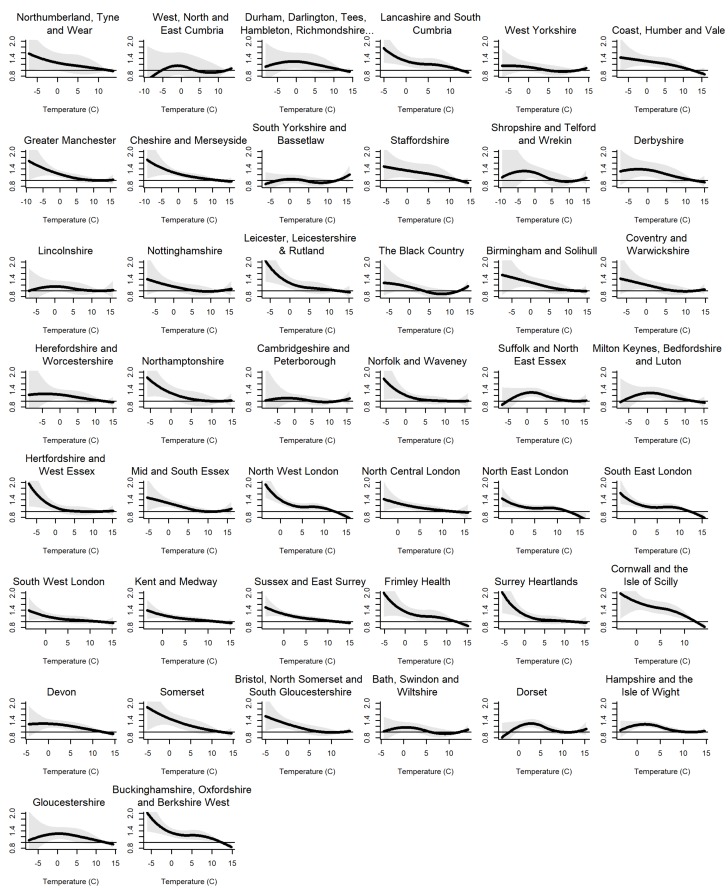
Cumulative association of temperature effect on mortality (November to March, by STP) for lags 0–28. The figures show the effect on all diseases, during 2007–2015.

There was a north-south disparity in daily mean temperatures, Cornwall and the Isles of Scilly STP had the highest median temperature (7.4 °C), and Northumberland, Tyne, and Wear STP the lowest (3.9 °C) (Table 1). The daily mean temperatures at the first percentile suggest colder winters during the earlier period than in the later period. For example, at a national level, temperature at the first percentile were −3.80 °C in the earlier period, compared to −2.0 °C in the second period. West, North and East Cumbria STP, which registered the lowest temperature at first percentile in the earlier period (−6.9 °C), was relatively milder in the late period (−2.4 °C). This pattern was consistent across all other areas. Figure 1 shows the temperature–mortality association across all STPs between 2007–2015.

**Figure 2:**
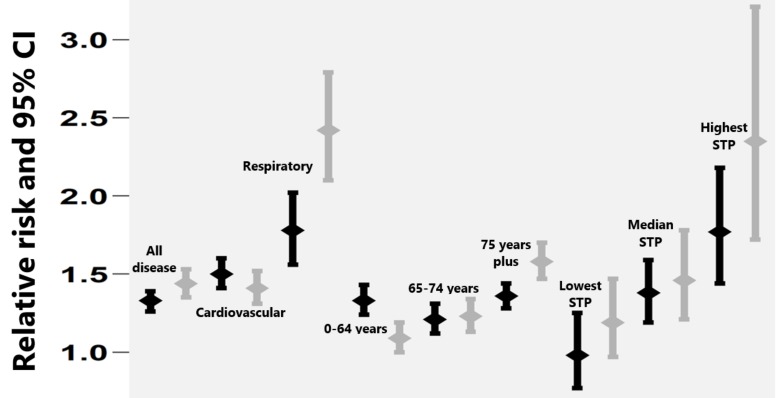
The mortality risk during the period before (black error bars) and after (grey bars) the CWP introduction. The error bars give the relative risk (95% CI) across all-diseases, disease specific, age-grouped, and for STPs with the lowest, median, and highest relative risk.

At a national level, cold risk was highest in the oldest age group 75+ years (RR 1.41, 1.35–1.47) and this significantly differed with risk at lower age groups; (RR 1.26, 1.20–1.33) and (RR 1.22, 1.16–1.29) for 0–64 years and 65–74 years, respectively. There was also a significant difference in cardiovascular (RR 1.50, 1.43–1.56) and respiratory mortality risk (RR 1.98, 1.80–2.17). Looking at the change in relative risk before and after CWP implementation showed a significant decline in mortality risk for those aged 0–64 years (RR 1.34, 1.23–1.45, to RR 1.09, 1.00–1.19), but a significant increase in mortality risk in those aged 75+ years (1.36, 1.28–1.44, to 1.58, 1.47–1.70), and in respiratory conditions (1.78, 1.56–2.02, to 2.4, 2.10–2.79) (Figure 2).

The difference in risk between the STP with the highest and lowest risk was also statistically significant (Somerset STP, RR 1.74, 1.44–2.09) and (Cambridgeshire and Peterborough STP, RR 1.19, 1.30–1.40) (Table 1). Furthermore, Somerset STP and Cornwall and the Isles of Scilly STP were the only two STPs whose risk levels were significantly greater than the England average (Table 1). Comparing risk between the two periods showed the steepest incline in risk was in Cornwall and the Isles of Scilly from RR (95% CI) 1.35, (1.04-1.76) in the early period, to RR (95% CI) 2.35 (1.72–3.22) in the late period. 

Heterogeneity tests show that the STP level cold risk varied more considerably in the late period (I^2^ = 72.1%), in comparison to the variation in risk in the earlier period (I^2^ = 56.2%). 

**Figure 3:**
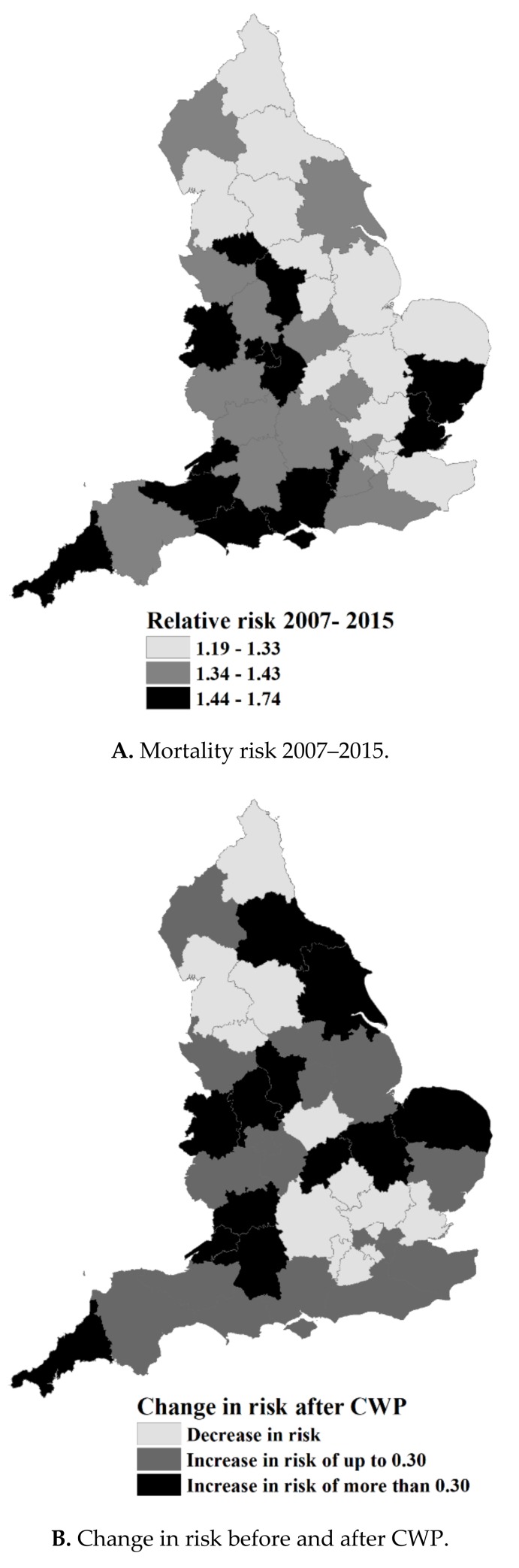

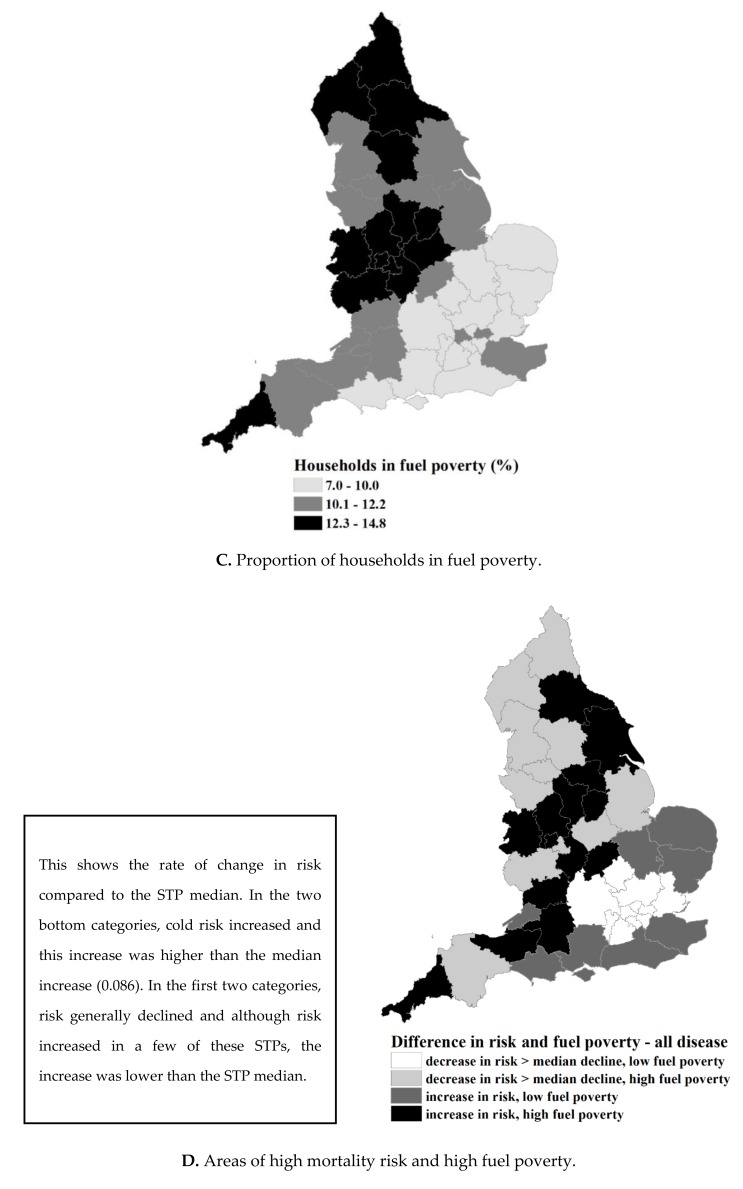
(**A**) The area distribution of mortality risk during 2007–2010, (**B**) the change in mortality risk between 2007–2010 and 2012–2015, (**C**) the cold weather mitigation score, and (**D**) areas of unmet need.

Mapping the area distribution in risk indicated the west and south west parts of England had higher levels of cold-related mortality (Figure 3A), and western and northern areas had a higher proportion of households experiencing fuel poverty (Figure 3C). Overlaying information on change in risk (Figure 3B), with information on fuel poverty (Figure 3C) found 14 STPs where mortality inclined at a higher rate than the median STP incline, and also had a higher than average experience of fuel poverty. These are: Durham, Darlington, Tees, Hambleton, Richmondshire and Whitby STP, Coast, Humber and Vale STP, South Yorkshire and Bassetlaw STP, Staffordshire STP, Shropshire and Telford and Wrekin STP, Derbyshire STP, Nottinghamshire STP, The Black Country STP, Coventry and Warwickshire STP, Northamptonshire STP, Cornwall and the Isles of Scilly STP, Somerset STP, Bath, Swindon and Wiltshire STP, and Gloucestershire STP (Table 1, Figure 3D). The sensitivity analysis (as described earlier) did not substantially change the observed spatial patterns; these results have been added to the supplementary material as (Table S1, Figure S2A,B,D).

## 4. Discussion

### 4.1. Main Finding of This Study 

This study highlights area variations in the mortality risk from cold exposure during winter months in England. It is the first study in England to systematically examine the change in mortality risk before and after implementation of the CWP, and to report the distribution and the change in risk at the level of STPs. 

Our findings show significant differences in risk between STPs with the highest and lowest risk. Additionally, mortality risk in the two STPs with the highest risk (Somerset and Cornwall and the Isles of Scilly) was significantly higher than the England level risk, despite milder winters. National level analysis found the highest risk in the oldest age group and for respiratory conditions. When comparing the national change in mortality risk before and after the CWP implementation, we observed a statistically significant drop in risk in the youngest age group and a statistically significant increase in the oldest group and in respiratory diseases. The heterogeneity test found variation in effect size by area increased considerably during the second. Lastly, combining area risk with fuel poverty statistics revealed 14 STPs where increase in risk surpassed the STP median increase and where fuel poverty was greater than the national average, suggesting that existing resources may not adequately address the adverse effects of cold exposures, particularly, in the identified 14 areas.

One previous study on mortality effect of outdoor temperatures in England found the East of England region had a significantly higher mortality risk, although regional differences diminished after years with missing pollution data were excluded [22]. Another study on the effect of indoor temperature found significant regional variations in excess winter deaths, whereby the West Midlands region had the highest excess mortality and London region had the lowest [11]. These studies may be criticized because regional analyses conceals the variation that occurs in smaller geographies. A district level study found uneven distribution in effect for those aged 75–84 years, whereby, men and women had a greater odds of death in the eastern and southwest parts of England, respectively [23].

Our study is the first to use STPs to demonstrate spatial variation in cold-related mortality. STPs geographies are large enough to enable the estimation of risk but small enough to discern spatial distribution at sub-regional levels. STPs have a role in strengthening prevention, early intervention, and integrating NHS and local authority services, making analysis at this level meaningful to local decision-makers. We found that STPs in the southern part of England had the highest mortality risk, despite the milder winters; this indicates that beyond environmental factors, social factors, such as differences in adaptation measures or in the implementation of public health interventions, may play a role in determining cold risk. Our evaluation of the impact of CWP on mortality risk found an incline in risk in over two-thirds of the STPs, although this was only significant in the Coast, Humber, and Vale STP. 

We found a significant decline in risk for the youngest age groups, which supports the evidence showing a decline in cold effect in the UK over the years (with the greatest decline in the youngest age group) [1]. This may be explained by a combination of factors ranging from milder winters [8] to influenza vaccination [1]. However, we also found a significant incline in risk in the eldest and thus the overall decline may be stymied by increase in the aging population who are more vulnerable to the cold, which is exacerbated by the increasing cost of energy [24] and austerity measures. The evidence linking a cold effect in the UK to social-economic status is inconsistent; on one hand, there is no clear increase in risk by deprivation [22,25], partly owing to the fact that social housing in the UK is often more energy efficient that other types of residences [11], but on the other hand, the risk of fuel poverty rises sharply with decrease in household income [24].

The higher wintertime health burden in the UK compared to countries with colder climates suggests that impacts are preventable [8]. The proportion of UK households that are unable to keep their homes adequately warm is higher than those in comparable European countries [26], and up to two-thirds of fuel poor households lack sufficient heating [27]. There are also suggestions that indoor temperature may be a more sensitive determinant of cold risk in countries with moderate winters, such as the UK [28,29]. Although data on indoor temperature is difficult to obtain, fuel poverty statistics may give an indication of household warmth [27].

Mortality and morbidity from cold winters is predictable and largely preventable [7]; CWP was introduced to prevent such harmful effects [30]. CWP has five levels of alert, the highest levels 3 and 4 are issued in response to severe cold weather and a major national emergency, respectively [31]. Whilst it is important to pay attention to the coldest days where alert levels are highest, more emphasis should be given to lower CWP alert levels 0 (Year-round planning) and 1 (Winter Preparedness and Action) as these have a greater impact of reducing mortality and morbidity burden [7]. CWP level 0 recommendations to tackle fuel poverty by improving households’ energy efficiencies may also have associated environmental co-benefits, since improving the residential thermal temperatures not only reduces domestic energy use, but may also help meet environmental policy targets on reducing greenhouse gases [32].

In this study, the extent of area variation in risk by STP increased in the period after CWP implementation, suggesting that it is possible that some of the mortality experienced in these areas maybe avoidable if CWPs were equally implemented across England. Government schemes that require local co-ordination, such as the CWP implementation or home energy efficiency interventions, may offer mitigation against the effects of cold exposure, but it unlikely that these initiatives are evenly adopted across the country. 

The success of public health interventions require collaboration between health, social care, and other departments such as environmental services. STPs in England are well-placed to integrate these services as part of their role in strengthening prevention and early intervention [33]. The variations in risk shown in this study may be partly indicative of disengagement between local services, which may be barrier to CWP implementation. A report looking at the development of STPs found poor engagement from local government, and that local government leaders were often unclear about their role in developing STP plans [34]. Local government involvement is imperative because housing and improving indoor thermal comfort is integral to mitigating a cold effect on health [11,22]. Additionally, the local implementation of CWP should not be a reactive response to extreme weather [7], but should be fully embedded in the public health agenda and steered by public health managers and not by emergency planners [7]. Importantly, there should be increased engagement with primary care, whose full involvement has been lacking in awareness and implementation of the CWP [7], and in the development of STP place-based plans [34].

### 4.2. Limitations of This Study

In absence of information on the local implementation, it is difficult to entirely attribute changes in risk to the CWP in England, which limits our understanding on the drivers of observed variations. Likewise, we were unable to obtain comprehensive data on housing energy efficiency. Following the recent Department for Energy and Climate Change (DECC’s) strategy to improve housing energy efficiency standards to at least a Band C rating [5], we would expect to see a reduction in cold mortality risk in future analysis. The observed area and temporal variation may be attributable to a number of factors that were not captured by our datasets, such as changes in the political and economic climate that may have impacted on service provision, access, and ability to respond to emergencies. The variations may also be explained by differences in age distribution and deprivation, as well as the indirect effect of influenza and air quality [35]. Winter mortality attributable to influenza in England has been shown to vary considerably by age groups [36,37]. There has also been temporal variation in air pollution across the study period [38]. Nevertheless, because the CWP implementation is to a certain extent applicable across most if not all of these factors (as described in the introduction), our findings are more likely related to the implementation of the CWP in its entirety rather than the sum of its parts.

This study benefited from access to STP data, enabling us to overcome the limitations associated with analysis at larger geographies such as masking small area variations. The analysis also benefited from having several years of data post the CWP implementation, enabling the comparison of risk before and after the intervention. 

Future research should examine the risk from exposure to indoor temperature, which may have a stronger association with health outcomes than outdoor temperatures [28,29]. Since implementation of CWP takes place across different agents, future research should also examine the exact circumstances that explain the area variation in cold risk. 

## 5. Conclusions

Previous studies on cold related mortality in England have found greater risk in the eldest [22], and there is mixed evidence on the effect modification by deprivation [8]. The literature on spatial variability is sparse. One study found higher mortality risk in the East of England region and postulated the relatively older population in this region as a possible mechanism [22]. Another study using higher spatial resolution found age- and sex-standardized cardio-respiratory mortality was highest in the North and West Midlands areas [23]. The Cold Weather Plan (CWP) for England was introduced in 2011 to prevent the adverse effect of cold exposure through local implementation. Its impact on health outcomes has not yet been evaluated.

To our knowledge, this is the first study in England to characterize cold-related mortality using the newly-formed Sustainability and Transformation Partnerships (STPs) geographies. We found STPs in the South West of England (Cornwall and Somerset) had higher mortality risk during the study period (2007–2015), despite having milder winters. A comparison of mortality risk in the period before and after the introduction of the CWP showed variation in mortality risk by area increased considerably during the second period. The national level analysis found a significant decline in cold-related mortality risk post CWP implementation in the youngest age group (0–64 years), but a significant increase in risk in the eldest group (75+ years) and from respiratory diseases. Lastly, combining area risk with fuel poverty statistics highlighted 14 STPs where the existing resources may be adequately address the adverse effects of cold exposures, and would therefore benefit from more targeted measures of intervention.

## Figures and Tables

**Table 1:** Distribution of daily mean temperatures at national and STP level, 2007–2015, mortality risk during winter months only (November–March); estimated by comparing temperature at the first percentile with the highest temperature. The table also shows the fraction of wintertime deaths attributable to low temperatures (Population Attributable Fraction (PAF)) and percentage of households experiencing fuel poverty in 2015.

Sustainability and Transformation Partnerships (STPs)	Lowest 2007–2015	First Percentile 2007–2015	Median 2007–2015	Highest 2007–2015	Relative Risk (95% CI) 2007–2015	PAF	Fuel Poverty (%) 2015
England	−7.00	−2.97	5.80	14.30	1.35 (1.30, 1.40)	14.05	11.04
Cambridgeshire and Peterborough (STP 21)	−8.53	−3.06	6.09	15.89	1.19 (1.01, 1.40)	8.58	8.14
Lincolnshire (STP 13)	−7.13	−2.72	5.58	15.82	1.23 (1.04, 1.45)	10.65	12.22
South Yorkshire and Bassetlaw (STP 9)	−6.55	−3.09	5.44	15.85	1.23 (1.09, 1.39)	10.81	11.60
North East London (STP 29)	−2.20	−1.03	7.35	16.42	1.25 (1.10, 1.42)	11.24	11.03
Norfolk and Waveney (STP 22)	−5.66	−1.47	5.99	15.90	1.26 (1.10, 1.46)	12.52	9.54
Hertfordshire and West Essex (STP 25)	−7.08	−3.04	5.60	15.25	1.28 (1.13, 1.46)	12.64	7.08
South West London (STP 31)	−3.95	−2.14	6.45	15.30	1.29 (1.12, 1.47)	12.29	9.13
Lancashire and South Cumbria (STP 4)	−5.01	−2.54	5.85	14.02	1.30 (1.18, 1.44)	12.66	12.07
Northumberland, Tyne, and Wear (STP 1)	−7.11	−3.31	3.94	13.78	1.30 (1.16, 1.46)	13.99	13.14
Durham, Darlington, Tees, Hambleton, Richmondshire, and Whitby (STP 3)	−8.29	−3.84	4.43	14.21	1.30 (1.14, 1.48)	13.49	13.21
West Yorkshire (STP 5)	−6.64	−2.13	5.41	14.55	1.31 (1.19, 1.44)	13.95	13.24
Nottinghamshire (STP 14)	−7.30	−2.60	5.75	15.68	1.31 (1.13, 1.51)	13.83	12.84
Kent and Medway (STP 32)	−3.51	−1.49	6.76	15.38	1.32 (1.18, 1.47)	13.58	10.05
South East London (STP 30)	−3.26	−1.63	7.08	15.65	1.33 (1.17, 1.50)	13.63	8.77
Herefordshire and Worcestershire (STP 19)	−8.93	−3.23	5.96	15.03	1.33 (1.14, 1.56)	13.60	13.48
Northamptonshire (STP 20)	−5.99	−3.33	5.40	14.98	1.33 (1.20, 1.48)	21.14	11.84
Milton Keynes, Bedfordshire, and Luton (STP 24)	−7.25	−3.07	6.02	15.28	1.34 (1.14, 1.58)	14.20	8.03
Buckinghamshire, Oxfordshire, and Berkshire West (STP 44)	−6.16	−2.94	5.94	14.93	1.34 (1.19, 1.51)	14.12	9.35
Bath, Swindon and Wiltshire (STP 40)	−4.43	−2.36	6.10	14.28	1.34 (1.16, 1.55)	13.75	11.42
Coast, Humber and Vale (STP 6)	−7.31	−2.88	5.42	15.89	1.35 (1.20, 1.53)	15.63	11.64
Leicester, Leicestershire and Rutland (STP 15)	−6.25	−2.31	5.81	15.33	1.36 (1.18, 1.58)	15.70	13.33
Cheshire and Merseyside (STP 8)	−9.21	−3.51	6.07	15.67	1.37 (1.24, 1.50)	14.97	11.30
Sussex and East Surrey (STP 33)	−4.39	−1.81	6.63	15.03	1.37 (1.24, 1.50)	14.90	9.37
Devon (STP 37)	−4.45	−1.59	6.84	14.62	1.38 (1.22, 1.56)	14.78	12.15
Gloucestershire (STP 43)	−7.41	−3.29	5.95	14.59	1.38 (1.17, 1.64)	14.88	11.40
North West London (STP 27)	−3.41	−1.79	6.82	15.98	1.39 (1.22, 1.59)	16.25	10.68
North Central London (STP 28)	−3.29	−1.56	6.93	15.93	1.42 (1.23, 1.64)	16.97	9.96
Surrey Heartlands (STP 35)	−5.75	−3.05	6.49	15.48	1.42 (1.20, 1.67)	16.16	7.69
West, North and East Cumbria (STP 2)	−9.31	−4.04	4.50	13.78	1.42 (1.14, 1.76)	16.99	12.26
Staffordshire (STP 10)	−5.69	−3.39	4.72	14.57	1.43 (1.25, 1.64)	18.17	12.48
Frimley Health (STP 34)	−5.31	−2.91	6.24	15.28	1.44 (1.19, 1.74)	16.92	8.77
Hampshire and the Isle of Wight (STP 42)	−3.76	−1.48	6.96	14.74	1.44 (1.31, 1.59)	16.86	9.76
Coventry and Warwickshire (STP 18)	−6.72	−2.98	5.73	14.97	1.44 (1.24, 1.67)	17.37	12.77
Greater Manchester (STP 7)	−9.21	−3.21	5.61	15.84	1.45 (1.31, 1.60)	18.54	12.00
Dorset (STP 41)	−3.89	−1.23	7.43	15.44	1.47 (1.26, 1.71)	17.68	10.02
The Black Country (STP 16)	−6.43	−2.85	5.46	14.79	1.48 (1.31, 1.67)	18.96	13.86
Bristol, North Somerset, and South Gloucestershire (STP 39)	−5.18	−2.38	6.42	14.25	1.48 (1.28, 1.70)	17.33	10.94
Shropshire and Telford and Wrekin (STP 11)	−9.64	−3.48	5.93	15.14	1.48 (1.22, 1.81)	18.12	14.79
Suffolk and North East Essex (STP 23)	−6.73	−2.57	5.81	15.63	1.49 (1.30, 1.71)	19.60	8.64
Birmingham and Solihull (STP 17)	−7.65	−3.15	5.60	14.95	1.50 (1.30, 1.72)	19.11	14.13
Derbyshire (STP 12)	−7.34	−3.43	4.93	15.00	1.50 (1.30, 1.73)	20.27	12.54
Mid and South Essex (STP 26)	−5.42	−2.07	6.36	16.20	1.57 (1.38, 1.80)	22.06	6.99
Cornwall and the Isles of Scilly (STP 36)	−3.49	−1.16	7.44	14.38	1.71 (1.44, 2.04)	22.14	14.29
Somerset (STP 38)	−5.37	−2.30	6.43	14.79	1.74 (1.44, 2.09)	24.31	12.24

## Supplementary Materials

The following are available online at http://www.mdpi.com/1660-4601/15/11/2588/s1, Figure S1: Size and location of the Sustainable and Transformation Partnerships in England, Figure S2: (A) the area distribution of mortality risk during 2007–2010, (B) the change in mortality risk between 2007–2010 and 2011–2015, and (D) areas of unmet need, Table S1. Distribution of daily mean temperatures at national and STP level, 2007-2015, mortality risk during winter months only (November–March); estimated by comparing temperature at 0 °C with temperature at 13 °C. The table also shows the fraction of wintertime deaths attributable to low temperatures (Population Attributable Fraction (PAF)).
