# Supplementary material for: Variation in Cold-Related Mortality in England Since the Introduction of the Cold Weather Plan: Which Areas Have the Greatest Unmet Needs?
Source: Int J Environ Res Public Health. 2018 Nov 19;15(11):2588. doi: 10.3390/ijerph15112588 (PMC6265768; doi:10.3390/ijerph15112588)
Supplement: Supplementary file 1 [file ijerph-15-02588-s001.pdf]

International Journal of
*Environmental Research
and Public Health*

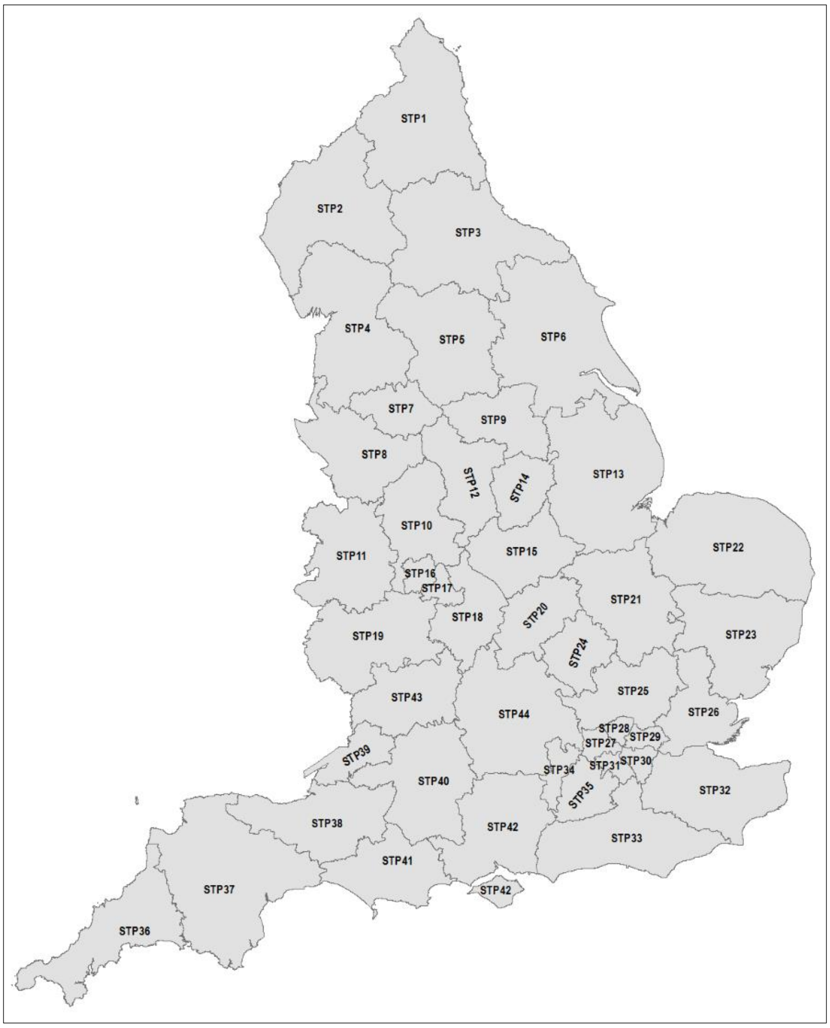

Figure S1. Size and location of the Sustainable and Transformation Partnerships in England.

Table S1. Distribution of daily mean temperatures at national and STP level, 2007-2015, mortality risk during winter months only (November–March); estimated by comparing temperature at 0 °C with temperature at 13 °C. The table also shows the fraction of wintertime deaths attributable to low temperatures (Population Attributable Fraction (PAF)).

**Sustainability Transformation Partnerships (STPs)**	**Relative risk (95% CI) 2007–2015**	**PAF**
England	1.25 (1.22–1.29)	10.92
Cambridgeshire and Peterborough	1.12 (1.00-1.26)	5.45
Lincolnshire	1.15 (1.03-1.30)	7.43
South Yorkshire and Bassetlaw	1.15 (1.06-1.25)	7.1
North East London	1.18 (1.07–1.30)	6.96
Norfolk and Waveney	1.19 (1.07–1.33)	8.66
Hertfordshire and West Essex	1.20 (1.09–1.31)	8.88
South West London	1.20 (1.09–1.33)	8.54
Durham, Darlington, Tees, Hambleton, Richmondshire and Whitby	1.21 (1.10–1.33)	10.13
Nottinghamshire	1.21 (1.09–1.34)	9.23
Northumberland, Tyne and Wear	1.22 (1.12–1.33)	11.12
Lancashire and South Cumbria	1.23 (1.14–1.33)	10.44
West Yorkshire	1.23 (1.14–1.33)	10.78
Herefordshire and Worcestershire	1.23 (1.10–1.38)	9.62
Milton Keynes, Bedfordshire and Luton	1.23 (1.10–1.38)	9.75
Coast, Humber and Vale	1.23 (1.13–1.34)	10.6
Cheshire and Merseyside	1.23 (1.16–1.32)	10.13
Kent and Medway	1.24 (1.14–1.35)	9.73
South East London	1.24 (1.12–1.36)	9.22
Buckinghamshire, Oxfordshire and Berkshire West	1.24 (1.13–1.35)	10.06
Bath, Swindon and Wiltshire	1.26 (1.12–1.41)	10.87
Leicester, Leicestershire and Rutland	1.26 (1.13–1.40)	11.09
Gloucestershire	1.26 (1.12–1.43)	10.85
Sussex and East Surrey	1.27 (1.18–1.37)	10.88
North West London	1.28 (1.16–1.40)	10.9
Surrey Heartlands	1.28 (1.14–1.44)	11.06
Devon	1.29 (1.17–1.43)	11.37
West, North and East Cumbria	1.29 (1.10–1.51)	12.98
Greater Manchester	1.29 (1.20–1.38)	12.36
North Central London	1.30 (1.17–1.45)	11.54
Staffordshire	1.30 (1.17–1.43)	13.21
Frimley Health	1.30 (1.13–1.49)	11.76
Coventry and Warwickshire	1.30 (1.17–1.45)	12.32
Shropshire and Telford and Wrekin	1.32 (1.15–1.51)	12.88
The Black Country	1.33 (1.22–1.46)	13.69
Suffolk and North East Essex	1.33 (1.20–1.47)	13.61
Birmingham and Solihull	1.33 (1.21–1.47)	13.64
Derbyshire	1.33 (1.21–1.48)	14.24
Hampshire and the Isle of Wight	1.34 (1.24–1.45)	12.99
Dorset	1.35 (1.20–1.52)	12.42
Bristol, North Somerset and South Gloucestershire	1.35 (1.21–1.52)	13.57
Northamptonshire	1.38 (1.22–1.55)	14.88
Mid and South Essex	1.38 (1.26–1.52)	14.76
Somerset	1.52 (1.32–1.75)	18.68
Cornwall and the Isles of Scilly	1.57 (1.35–1.81)	17.87

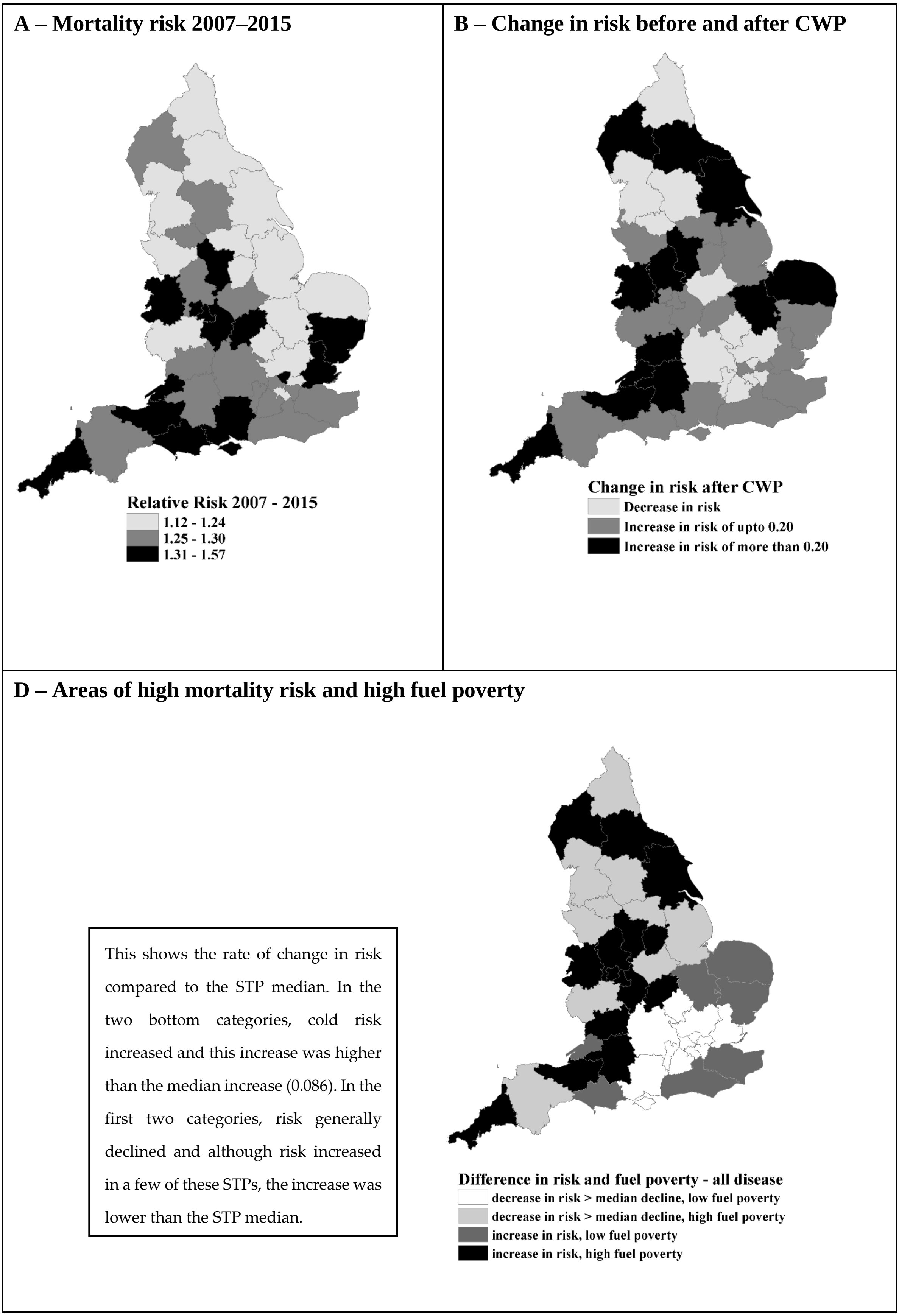

Figure S2. (**A**) the area distribution of mortality risk during 2007–2010, (**B**) the change in mortality risk between 2007–2010 and 2011–2015, and (**D**) areas of unmet need.
